# Suggestive genetic association between age-related biomarkers and hearing impairment: a mendelian randomization study

**DOI:** 10.1016/j.bjorl.2026.101918

**Published:** 2026-10-31

**Authors:** Xiaojun Jiang, Chen Tang, Wei Yuan

**Affiliations:** a Chongqing Medical University, Department of Otolaryngology, Chongqing, China; b Chongqing University, School of Medicine, Renji Hospital, Department of Otolaryngology, Chongqing, China; c The Fifth People's Hospital of Chongqing, Chongqing, China; d Department of Otolaryngology, Chongqing People's Hospital, Chongqing, China

**Keywords:** Mendelian randomization, Epigenetic age, Telomere length, Hearing impairment, Inflammation

## Abstract

**Objective:**

Observational studies suggest links between biological aging biomarkers and hearing impairment, but causal inference remains limited. Mendelian Randomization (MR) offers a genetic epidemiological approach to explore potential causal relationships. We used MR to evaluate potential causal associations between various aging biomarkers and five subtypes of hearing impairment.

**Methods:**

We conducted a two-sample MR study using summary data from large-scale, European-ancestry genome-wide association studies. Biological aging biomarkers, including epigenetic clocks, overall and cell-specific telomere length, were used as exposures. Outcomes were age-related hearing impairment and four other subtypes. The inverse variance weighted method was our primary analysis, supported by comprehensive sensitivity analyses to assess result stability.

**Results:**

Our analysis observed nominal associations related to immune cell subtypes. Genetically predicted longer granulocyte telomere length was nominally associated with lower odds of mixed hearing loss (OR = 0.84, 95% CI 0.74‒0.96, p = 0.009), whereas longer leukocyte telomere length was nominally associated with higher odds of mixed hearing loss (OR = 1.21, 95% CI: 1.03–1.42, p = 0.02). Furthermore, a higher proportion of granulocytes was nominally associated with lower odds of age-related hearing impairment (OR = 0.72, 95% CI: 0.55‒0.95, p = 0.02). Although sensitivity analyses did not indicate substantial heterogeneity and horizontal pleiotropy, these associations should be interpreted with caution due to the lack of robust FDR-corrected evidence.

**Conclusion:**

Our findings suggest a complex landscape of cell-type specific and paradoxical associations. This may help shift attention from general anti-aging interventions towards dissecting specific cellular pathways for hearing health.

**Level of Evidence:**

This two-sample Mendelian randomization study provides genetic evidence suggesting potential associations between age-related biomarkers and hearing impairment, complementing findings from conventional observational studies.

## Highlights

• Longer granulocyte telomere length protects against mixed hearing loss (OR = 0.84).
• Longer leukocyte telomere length increases risk of hearing impairment (OR = 1.21).
• Higher granulocyte proportion reduces age-related hearing impairment risk.

## Introduction

Hearing impairment represents a formidable global health challenge, with recent estimates from the World Health Organization indicating that it affects over 1.5 billion people worldwide.^1^^,^^2^ A growing body of evidence has unequivocally linked hearing loss to a spectrum of devastating adverse outcomes, most notably accelerated cognitive decline and an increased risk of dementia, as well as social isolation and a severely diminished quality of life.^1^ Among its various forms, Age-Related Hearing Impairment (ARHI) is by far the most prevalent, representing an almost inevitable consequence of human aging. However, chronological age alone is a crude and often misleading predictor of its onset and progression. The specific biological aging processes ‒ the molecular and cellular drivers ‒ that orchestrate the degeneration of the auditory system remain poorly understood, representing a critical gap in our knowledge.^3^

Recent research has focused on molecular biomarkers of biological aging. Among the most promising are epigenetic clocks, which are predictive models that estimate biological age based on patterns of DNA methylation ‒ chemical modifications that accumulate on the genome throughout life. These clocks, such as the advanced GrimAge and PhenoAge, offer a more accurate measure of an individual's physiological decline than chronological age and have been strongly associated with various age-related diseases.^4^^,^^5^

Observational studies have begun to suggest a link between accelerated epigenetic aging and hearing loss.^6^ Similarly, telomere shortening ‒ a hallmark of cellular aging ‒ and associated chronic inflammation are hypothesized to contribute to cochlear damage.^7^^,^^8^ However, these observational findings are prone to confounding and reverse causality, making it difficult to establish true causal relationships.

Mendelian Randomization (MR) offers a genetic epidemiological approach to explore potential causal relationships by using genetic variants as proxies for an exposure.^9^ While previous MR studies have investigated these links, their results have been inconsistent and have several limitations. Most studies did not provide consistent evidence for an association between overall telomere length with ARHI and reported mixed findings for epigenetic clocks.^10^^,^^11^ These discrepancies may stem from methodological choices, such as treating hearing loss as a single entity, which overlooks the distinct pathologies of its subtypes, and using bulk tissue measures, which may mask the cell-type-specific signals of aging biomarkers.

## Methods

### Study design

This two-sample MR study was designed and reported in accordance with the MR-STROBE statement, a guideline developed to ensure transparent and comprehensive reporting of MR findings.^12^

To systematically investigate potential causal associations between biological aging and auditory dysfunction, we implemented a two-sample MR study (Fig. 1). This approach leverages genetic variants, identified from Genome-Wide Association Studys (GWAS), as Instrumental Variables (IVs) to proxy for the exposures. The validity of our MR analysis relies on three core assumptions: (i) The genetic variants are robustly associated with the exposure of interest (the relevance assumption); (ii) The variants are not associated with any confounders of the exposure-outcome relationship (the independence assumption); and (iii) The variants affect the outcome exclusively through their effect on the exposure (the exclusion assumption). By adhering to this framework and utilizing independent datasets for exposures and outcomes, our study design aims to provide new genetic evidence for potential causal role of epigenetic biomarkers in the etiology of specific hearing impairment subtypes.

**Fig. 1:**
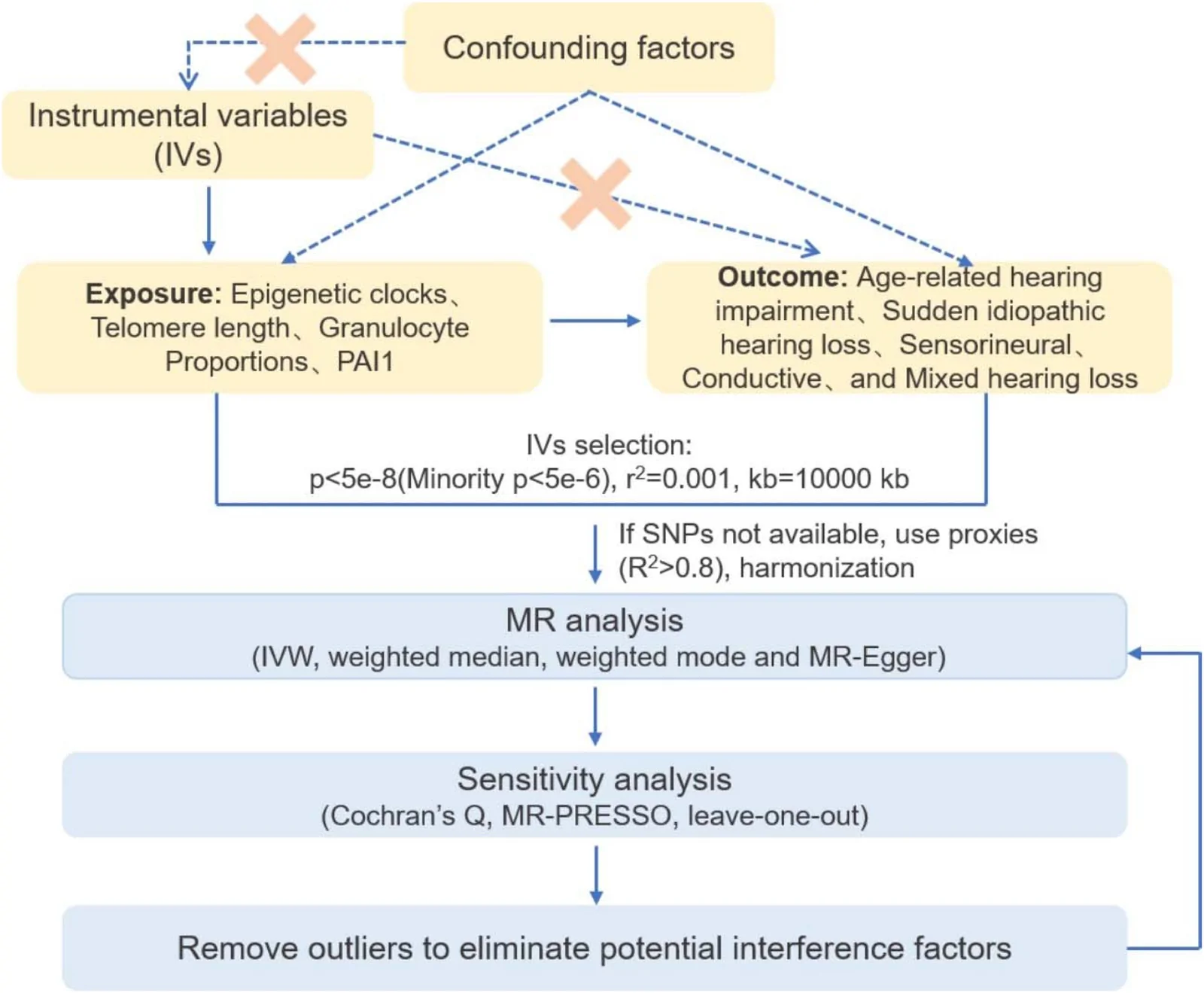
Overall study design of the MR analysis.

### Data sources

This study used publicly available GWAS aggregated data. The use of aggregated non personal data eliminates the need for additional institutional review committee approval for this secondary analysis. And all analyses are strictly limited to populations of European descent to ensure the robustness of our causal reasoning.

Exposure data for four epigenetic clocks (PhenoAge, GrimAge, HannumAge, HorvathAge) and two related biomarkers (granulocyte proportions, PAI1) were obtained from a GWAS meta-analysis with sample sizes up to 34,470 individuals.^13^ For telomere length, we obtained aggregated statistical data on overall and white blood cell telomere length from a large-scale study at the UK Biobank, with a sample size of 472174 individuals.^14^ Data for cell-specific telomere length (B-cells, granulocytes, lymphocytes, memory T-cells, naive T-cells, and NK cells) were derived from a separate study of 902 Dutch individuals.^15^

Outcome data for five hearing impairment subtypes were sourced from two large consortia, with phenotype definitions based on self-reported hearing-related traits in the UK Biobank and registry-based ICD-10 diagnostic codes in FinnGen. Summary statistics for age-related hearing impairment were obtained from the UK Biobank, with a sample size of 330759 individuals (GWAS ID: ebi-a-GCST90012115). Data for four other subtypes ‒ sudden idiopathic hearing loss (3861 cases), sensorineural hearing loss (44,745 cases), conductive hearing loss (3255 cases), and mixed conductive and sensorineural hearing loss (4810 cases) ‒ were obtained from the FinnGen consortium (Release R12), which included up to 437,331 controls. Detailed characteristics of each source GWAS dataset are summarized in Supplementary Table 1, including the GWAS ID, source, sample sizes, phenotype definition, diagnostic codes where applicable, and data source.

### Instrumental variable (IV) selection

To ensure the validity of IVs, a multi-step selection process was implemented. First, Single Nucleotide Polymorphisms (SNPs) associated with each exposure were identified at a genome-wide significance threshold of p < 5 × 10^−8^. This threshold was relaxed to p < 5 × 10^–6^ or p < 1 × 10^–5^ for exposures with a limited number of significant variants, to ensure that a sufficient number of SNPs can be screened for sensitivity analysis.^16^ Next, to ensure the independence of IVs, we performed Linkage Disequilibrium (LD) clumping using a strict threshold (*R^2^* < 0.001 within a 10,000 kb window). SNPs with a Minor Allele Frequency (MAF) below 0.01 and ambiguous palindromic SNPs were excluded. For IVs unavailable in the outcome GWAS, we identified proxy SNPs with high LD (*R^2^* > 0.8). Finally, the strength of each IV was assessed using the F-statistic, calculated as F = R^2^×(N-2)/(1-R²). Only IVs with an F-statistic > 10 were retained to minimize weak instrument bias.

### MR analysis

The primary causal association were assessed using the random-effects Inverse-Variance Weighted (IVW) method. The IVW method provides the highest statistical power by meta-analyzing the Wald ratios of each IV. To assess the reliability of findings and test for potential violations of this assumption, these primary results were complemented by three sensitivity analyses. We employed MR-Egger regression, which can detect and adjust for directional pleiotropy via its intercept term.^17^ Additionally, we used the weighted median method, which provides a consistent estimate if at least 50% of the weight comes from valid instruments, and the weighted mode method, which is robust to a plurality of invalid instruments.^18^ All statistical tests were two-tailed and performed in *R* (version 4.0.5) using the “TwoSampleMR” package. To control for multiple comparisons, False Discovery Rate (FDR) correction was applied using the Benjamini–Hochberg procedure.

### Sensitivity analyses

In sensitivity analyses, we first used Cochran’s Q test to assess heterogeneity among SNP-specific estimates; significant heterogeneity may suggest variability across instruments and potential horizontal pleiotropy. We then used MR-Egger regression to estimate directional horizontal pleiotropy via its intercept term. The weighted median and weighted mode methods were also employed, as they can provide more robust estimates in the presence of invalid instruments. The MR-PRESSO (Pleiotropy RESidual Sum and Outlier) test was used to identify and correct for the influence of potential outlier variants. Finally, to ensure no single SNP was disproportionately driving the association, a leave-one-out analysis was performed by iteratively removing one SNP at a time and re-estimating the causal link.

## Results

### Selection of instrumental variables

Following a multi-step filtering process based on genome-wide significance (with relaxed thresholds for exposures with fewer SNPs), linkage disequilibrium (*R*^2^ < 0.001), and minor allele frequency, we identified a set of independent genetic variants for each exposure. The number of IVs for each exposure ranged from 5 (PAI1) to 196 (leukocyte telomere length). The strength of these IVs was supported by F-statistics exceeding 10 for all individual SNPs (ranging from 19.74 to 1628.81), suggesting a low risk of weak instrument bias. Supplementary Table 2 provides a detailed list of instrumental variables, including F-statistics, *R*^2^ values, and proxy SNP information for each genetic variation.

### MR analysis

In our primary analysis using the IVW method, we identified four suggestive associations (p < 0.05) (Fig. 2). For ARHI, we observed two protective nominal associations. Genetically predicted higher granulocyte proportions were nominally associated with lower odds of ARHI. Specifically, a one-standard-deviation increase in granulocyte proportions was associated with a 28% reduction in the odds of ARHI (OR = 0.72, 95% CI 0.55–0.95, p = 0.021, FDR_p = 0.529). Similarly, genetically predicted longer B-cell telomere length was nominally associated with lower odds of ARHI, where each standard deviation increase in length was associated with a 2% decrease in the odds of ARHI (OR = 0.98, 95% CI 0.97–1.00, p = 0.03, FDR_p = 0.506). For mixed conductive and sensorineural hearing loss, we observed two paradoxical suggestive associations. Genetically predicted longer granulocyte telomere length was nominally associated with lower odds of mixed hearing loss. Each standard deviation increase in its length was associated with 16% lower odds of this hearing loss subtype (OR = 0.84, 95% CI 0.74–0.96, p = 0.009, FDR_p = 0.506). Conversely, genetically predicted longer leukocyte telomere length was nominally associated with higher odds of mixed hearing loss. Each standard deviation increase in its length was associated with 21% higher odds of mixed hearing loss (OR = 1.21, 95% CI 1.03–1.42, p = 0.022, FDR_p = 0.506). After FDR correction, none of these associations remained statistically significant; therefore, these findings should be interpreted with caution. No other exposures, including any of the epigenetic clocks, showed a nominal significant suggestive association with any hearing impairment subtype in the primary analysis. The full results of the primary analysis are presented in Table 1.

**Fig. 2:**
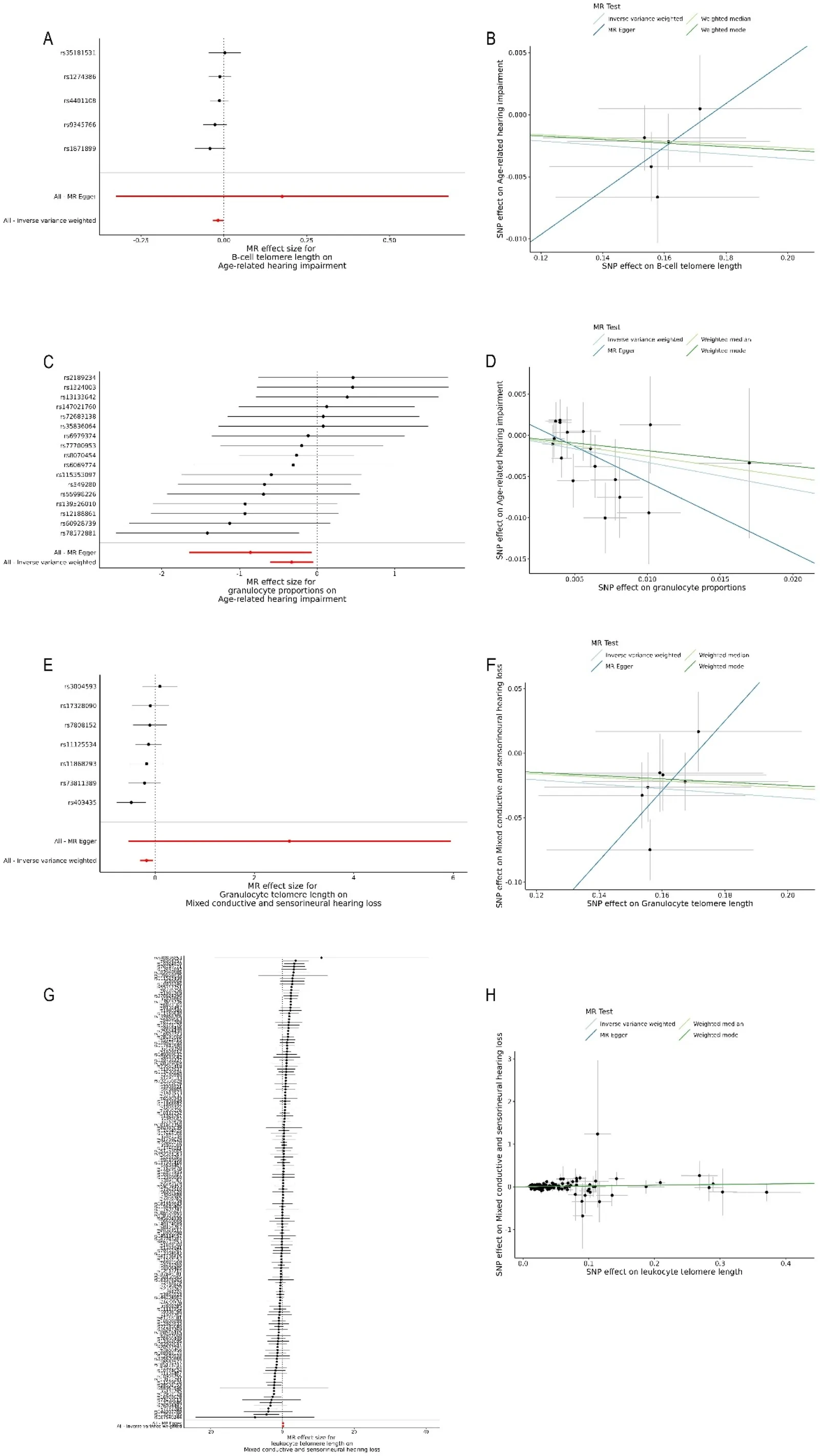
Primary analysis ‒ causal effect plots. (A) Forest plot of the causal association between B-cell telomere length and age-related hearing impairment. (B) Scatter plot illustrating the causal effect of B-cell telomere length on age-related hearing impairment using different MR methods. (C) Forest plot of the causal association between granulocyte proportions and age-related hearing impairment. (D) Scatter plot illustrating the causal effect of granulocyte proportions on age-related hearing impairment. (E) Forest plot of the causal association between granulocyte telomere length and mixed conductive and sensorineural hearing loss. (F) Scatter plot illustrating the causal effect of granulocyte telomere length on mixed conductive and sensorineural hearing loss. (G) Forest plot of the causal association between leukocyte telomere length and mixed conductive and sensorineural hearing loss. (H) Scatter plot illustrating the causal effect of leukocyte telomere length on mixed conductive and sensorineural hearing loss.

**Table 1:** Causal estimates for the associations between genetically predicted biomarkers and hearing impairment (primary analysis, before outlier removal).

Exposure	Outcome	N.SNPs	Methods	OR (95% CI)	p	FDR
B-cell telomere length	Age-related hearing impairment	5	IVW	0.9825 (0.9669‒0.9983)	0.03	0.529
B-cell telomere length	Age-related hearing impairment	5	MR Egger	1.1925 (0.722–1.9695)	0.541	0.84
B-cell telomere length	Age-related hearing impairment	5	Weighted median	0.9867 (0.967–1.0069)	0.197	0.629
B-cell telomere length	Age-related hearing impairment	5	Weighted mode	0.9858 (0.9627–1.0095)	0.304	0.826
B-cell telomere length	Conductive hearing loss, unspecified	5	IVW	1.0585 (0.8557–1.3092)	0.6	0.913
B-cell telomere length	Conductive hearing loss, unspecified	5	MR Egger	0.1718 (1e-04‒258.2109)	0.669	0.901
B-cell telomere length	Conductive hearing loss, unspecified	5	Weighted median	1.1792 (0.9325–1.4912)	0.169	0.629
B-cell telomere length	Conductive hearing loss, unspecified	5	Weighted mode	1.2914 (0.8681–1.9212)	0.275	0.826
B-cell telomere length	Mixed conductive and sensorineural hearing loss	5	IVW	1.015 (0.8793–1.1715)	0.839	0.947
B-cell telomere length	Mixed conductive and sensorineural hearing loss	5	MR Egger	14.1699 (0.1674–1199.7535)	0.326	0.84
B-cell telomere length	Mixed conductive and sensorineural hearing loss	5	Weighted median	0.9766 (0.8149–1.1704)	0.797	0.949
B-cell telomere length	Mixed conductive and sensorineural hearing loss	5	Weighted mode	0.9631 (0.7913–1.1722)	0.727	0.891
B-cell telomere length	Sensorineural hearing loss	5	IVW	1.0083 (0.9576–1.0617)	0.752	0.913
B-cell telomere length	Sensorineural hearing loss	5	MR Egger	0.5427 (0.0988–2.9815)	0.533	0.84
B-cell telomere length	Sensorineural hearing loss	5	Weighted median	0.984 (0.9205–1.052)	0.636	0.949
B-cell telomere length	Sensorineural hearing loss	5	Weighted mode	0.9805 (0.9069–1.0601)	0.648	0.889
B-cell telomere length	Sudden idiopathic hearing loss	5	IVW	0.9642 (0.774–1.201)	0.745	0.913
B-cell telomere length	Sudden idiopathic hearing loss	5	MR Egger	0.0073 (2.8e-05‒1.9084)	0.182	0.779
B-cell telomere length	Sudden idiopathic hearing loss	5	Weighted median	0.8552 (0.6818–1.0727)	0.176	0.629
B-cell telomere length	Sudden idiopathic hearing loss	5	Weighted mode	0.7994 (0.5309–1.2036)	0.344	0.826
Granulocyte proportions	Age-related hearing impairment	17	IVW	0.7203 (0.5453‒0.9513)	0.021	0.506
Granulocyte proportions	Age-related hearing impairment	17	MR Egger	0.4234 (0.1921‒0.9333)	0.05	0.721
Granulocyte proportions	Age-related hearing impairment	17	Weighted median	0.7763 (0.5251–1.1476)	0.204	0.629
Granulocyte proportions	Age-related hearing impairment	17	Weighted mode	0.8296 (0.4475–1.538)	0.561	0.889
Granulocyte proportions	Conductive hearing loss, unspecified	17	IVW	1.0268 (0.0407–25.8889)	0.987	0.998
Granulocyte proportions	Conductive hearing loss, unspecified	17	MR Egger	14.2517 (0.0016–127806.0744)	0.576	0.84
Granulocyte proportions	Conductive hearing loss, unspecified	17	Weighted median	2.7053 (0.0329–222.4454)	0.658	0.949
Granulocyte proportions	Conductive hearing loss, unspecified	17	Weighted mode	5.627 (0.0121–2606.7833)	0.589	0.889
Granulocyte proportions	Mixed conductive and sensorineural hearing loss	17	IVW	0.455 (0.0115–18.032)	0.675	0.913
Granulocyte proportions	Mixed conductive and sensorineural hearing loss	17	MR Egger	0.0836 (2.4e-06‒2860.544)	0.648	0.896
Granulocyte proportions	Mixed conductive and sensorineural hearing loss	17	Weighted median	0.4098 (0.0071–23.4835)	0.666	0.949
Granulocyte proportions	Mixed conductive and sensorineural hearing loss	17	Weighted mode	0.2052 (4e-04‒120.2157)	0.633	0.889
Granulocyte proportions	Sensorineural hearing loss	17	IVW	2.8974 (0.9504–8.8331)	0.061	0.716
Granulocyte proportions	Sensorineural hearing loss	17	MR Egger	5.6746 (0.239–134.711)	0.3	0.818
Granulocyte proportions	Sensorineural hearing loss	17	Weighted median	2.0152 (0.5476–7.4158)	0.292	0.694
Granulocyte proportions	Sensorineural hearing loss	17	Weighted mode	1.7681 (0.2755–11.345)	0.556	0.889
Granulocyte proportions	Sudden idiopathic hearing loss	17	IVW	1.679 (0.0564–49.9719)	0.765	0.913
Granulocyte proportions	Sudden idiopathic hearing loss	17	MR Egger	5.4415 (4e-04‒83643.4758)	0.735	0.915
Granulocyte proportions	Sudden idiopathic hearing loss	17	Weighted median	0.8111 (0.0125–52.6462)	0.922	0.977
Granulocyte proportions	Sudden idiopathic hearing loss	17	Weighted mode	60.1609 (0.0134–270563.371)	0.354	0.826
Granulocyte telomere length	Age-related hearing impairment	7	IVW	1.002 (0.9888–1.0153)	0.769	0.913
Granulocyte telomere length	Age-related hearing impairment	7	MR Egger	0.9097 (0.6485–1.276)	0.607	0.867
Granulocyte telomere length	Age-related hearing impairment	7	Weighted median	1.0022 (0.9851–1.0196)	0.8	0.949
Granulocyte telomere length	Age-related hearing impairment	7	Weighted mode	1.0039 (0.98–1.0284)	0.762	0.891
Granulocyte telomere length	Conductive hearing loss, unspecified	7	IVW	0.8876 (0.7664–1.028)	0.111	0.801
Granulocyte telomere length	Conductive hearing loss, unspecified	7	MR Egger	1.314 (0.026–66.5288)	0.897	0.946
Granulocyte telomere length	Conductive hearing loss, unspecified	7	Weighted median	0.8779 (0.7281–1.0584)	0.172	0.629
Granulocyte telomere length	Conductive hearing loss, unspecified	7	Weighted mode	0.9045 (0.6837–1.1965)	0.508	0.889
Granulocyte telomere length	Mixed conductive and sensorineural hearing loss	7	IVW	0.8419 (0.7394‒0.9586)	0.009	0.506
Granulocyte telomere length	Mixed conductive and sensorineural hearing loss	7	MR Egger	14.9634 (0.5831–384.0087)	0.163	0.761
Granulocyte telomere length	Mixed conductive and sensorineural hearing loss	7	Weighted median	0.874 (0.7433–1.0277)	0.103	0.629
Granulocyte telomere length	Mixed conductive and sensorineural hearing loss	7	Weighted mode	0.8828 (0.7056–1.1044)	0.317	0.826
Granulocyte telomere length	Sensorineural hearing loss	7	IVW	0.9596 (0.9128–1.0088)	0.106	0.801
Granulocyte telomere length	Sensorineural hearing loss	7	MR Egger	0.3586 (0.1101–1.1682)	0.149	0.747
Granulocyte telomere length	Sensorineural hearing loss	7	Weighted median	0.9539 (0.8977–1.0136)	0.128	0.629
Granulocyte telomere length	Sensorineural hearing loss	7	Weighted mode	0.9399 (0.8451–1.0454)	0.297	0.826
Granulocyte telomere length	Sudden idiopathic hearing loss	7	IVW	1.0572 (0.9235–1.2102)	0.42	0.89
Granulocyte telomere length	Sudden idiopathic hearing loss	7	MR Egger	0.7169 (0.0194–26.5197)	0.864	0.945
Granulocyte telomere length	Sudden idiopathic hearing loss	7	Weighted median	0.9859 (0.8279–1.174)	0.873	0.966
Granulocyte telomere length	Sudden idiopathic hearing loss	7	Weighted mode	0.9491 (0.7329–1.2291)	0.706	0.891
Grimage	Age-related hearing impairment	26	IVW	0.9961 (0.9905–1.0018)	0.177	0.801
Grimage	Age-related hearing impairment	26	MR Egger	0.9994 (0.9834–1.0157)	0.942	0.954
Grimage	Age-related hearing impairment	26	Weighted median	0.9975 (0.9898–1.0052)	0.519	0.886
Grimage	Age-related hearing impairment	26	Weighted mode	1.0006 (0.9832–1.0182)	0.948	0.97
Grimage	Conductive hearing loss, unspecified	26	IVW	0.9792 (0.9106–1.0529)	0.571	0.913
Grimage	Conductive hearing loss, unspecified	26	MR Egger	0.9222 (0.7776–1.0937)	0.361	0.84
Grimage	Conductive hearing loss, unspecified	26	Weighted median	0.9869 (0.9066–1.0744)	0.761	0.949
Grimage	Conductive hearing loss, unspecified	26	Weighted mode	1.0333 (0.9159–1.1656)	0.599	0.889
Grimage	Mixed conductive and sensorineural hearing loss	26	IVW	0.9819 (0.9367–1.0293)	0.448	0.89
Grimage	Mixed conductive and sensorineural hearing loss	26	MR Egger	0.9074 (0.8127–1.0131)	0.097	0.721
Grimage	Mixed conductive and sensorineural hearing loss	26	Weighted median	0.9616 (0.8965–1.0314)	0.273	0.694
Grimage	Mixed conductive and sensorineural hearing loss	26	Weighted mode	0.9293 (0.8294–1.0412)	0.218	0.826
Grimage	Sensorineural hearing loss	26	IVW	0.9978 (0.9754–1.0207)	0.848	0.947
Grimage	Sensorineural hearing loss	26	MR Egger	0.9579 (0.9103–1.008)	0.111	0.721
Grimage	Sensorineural hearing loss	26	Weighted median	0.9866 (0.9616–1.0122)	0.301	0.694
Grimage	Sensorineural hearing loss	26	Weighted mode	0.9827 (0.9504–1.0162)	0.319	0.826
Grimage	Sudden idiopathic hearing loss	26	IVW	1.0423 (0.9775–1.1113)	0.206	0.801
Grimage	Sudden idiopathic hearing loss	26	MR Egger	1.0267 (0.8808–1.1969)	0.739	0.915
Grimage	Sudden idiopathic hearing loss	26	Weighted median	1.0333 (0.9495–1.1244)	0.448	0.837
Grimage	Sudden idiopathic hearing loss	26	Weighted mode	1.0293 (0.924–1.1467)	0.605	0.889
Hannumage	Age-related hearing impairment	9	IVW	0.9973 (0.9857–1.009)	0.65	0.913
Hannumage	Age-related hearing impairment	9	MR Egger	1.0268 (0.9857–1.0695)	0.245	0.818
Hannumage	Age-related hearing impairment	9	Weighted median	0.9983 (0.9884–1.0083)	0.743	0.949
Hannumage	Age-related hearing impairment	9	Weighted mode	0.9988 (0.9866–1.0112)	0.856	0.936
Hannumage	Conductive hearing loss, unspecified	9	IVW	0.9778 (0.8918–1.0722)	0.633	0.913
Hannumage	Conductive hearing loss, unspecified	9	MR Egger	0.8793 (0.6077–1.2721)	0.517	0.84
Hannumage	Conductive hearing loss, unspecified	9	Weighted median	0.9982 (0.8885–1.1214)	0.976	0.977
Hannumage	Conductive hearing loss, unspecified	9	Weighted mode	1.0198 (0.8437–1.2326)	0.845	0.936
Hannumage	Mixed conductive and sensorineural hearing loss	9	IVW	1.049 (0.9571–1.1497)	0.307	0.833
Hannumage	Mixed conductive and sensorineural hearing loss	9	MR Egger	0.8529 (0.6067–1.199)	0.39	0.84
Hannumage	Mixed conductive and sensorineural hearing loss	9	Weighted median	1.0148 (0.9209–1.1183)	0.767	0.949
Hannumage	Mixed conductive and sensorineural hearing loss	9	Weighted mode	0.9789 (0.866–1.1065)	0.742	0.891
Hannumage	Sensorineural hearing loss	9	IVW	1.0044 (0.9805–1.0288)	0.722	0.913
Hannumage	Sensorineural hearing loss	9	MR Egger	0.989 (0.9018–1.0845)	0.82	0.931
Hannumage	Sensorineural hearing loss	9	Weighted median	0.9866 (0.9558–1.0183)	0.403	0.783
Hannumage	Sensorineural hearing loss	9	Weighted mode	0.9813 (0.9391–1.0255)	0.426	0.889
Hannumage	Sudden idiopathic hearing loss	9	IVW	1.0322 (0.9511–1.1201)	0.448	0.89
Hannumage	Sudden idiopathic hearing loss	9	MR Egger	1.1533 (0.8335–1.5959)	0.418	0.84
Hannumage	Sudden idiopathic hearing loss	9	Weighted median	1.049 (0.9466–1.1625)	0.361	0.722
Hannumage	Sudden idiopathic hearing loss	9	Weighted mode	1.0635 (0.914–1.2373)	0.449	0.889
Horvathage	Age-related hearing impairment	23	IVW	1.0034 (0.9989–1.008)	0.141	0.801
Horvathage	Age-related hearing impairment	23	MR Egger	1.0048 (0.9929–1.0169)	0.437	0.84
Horvathage	Age-related hearing impairment	23	Weighted median	1.0068 (1.0008–1.0128)	0.026	0.629
Horvathage	Age-related hearing impairment	23	Weighted mode	1.0074 (1.0001–1.0148)	0.059	0.826
Horvathage	Conductive hearing loss, unspecified	24	IVW	0.9767 (0.9344–1.0209)	0.297	0.833
Horvathage	Conductive hearing loss, unspecified	24	MR Egger	1.0343 (0.9325–1.1473)	0.53	0.84
Horvathage	Conductive hearing loss, unspecified	24	Weighted median	0.9949 (0.9385–1.0548)	0.864	0.966
Horvathage	Conductive hearing loss, unspecified	24	Weighted mode	1.0532 (0.9616–1.1534)	0.276	0.826
Horvathage	Mixed conductive and sensorineural hearing loss	24	IVW	0.9893 (0.9555–1.0244)	0.545	0.908
Horvathage	Mixed conductive and sensorineural hearing loss	24	MR Egger	1.0123 (0.9323–1.0991)	0.774	0.915
Horvathage	Mixed conductive and sensorineural hearing loss	24	Weighted median	0.9989 (0.9517–1.0485)	0.965	0.977
Horvathage	Mixed conductive and sensorineural hearing loss	24	Weighted mode	1.0305 (0.9557–1.1111)	0.443	0.889
Horvathage	Sensorineural hearing loss	24	IVW	1.0054 (0.9911–1.02)	0.458	0.89
Horvathage	Sensorineural hearing loss	24	MR Egger	1.0278 (0.9944–1.0624)	0.118	0.721
Horvathage	Sensorineural hearing loss	24	Weighted median	1.0127 (0.9943–1.0314)	0.177	0.629
Horvathage	Sensorineural hearing loss	24	Weighted mode	1.0174 (0.9927–1.0427)	0.182	0.826
Horvathage	Sudden idiopathic hearing loss	24	IVW	1.0196 (0.9808–1.0599)	0.327	0.833
Horvathage	Sudden idiopathic hearing loss	24	MR Egger	0.9847 (0.8982–1.0795)	0.745	0.915
Horvathage	Sudden idiopathic hearing loss	24	Weighted median	1.0072 (0.9539–1.0634)	0.796	0.949
Horvathage	Sudden idiopathic hearing loss	24	Weighted mode	0.9982 (0.9338–1.067)	0.958	0.97
Leukocyte telomere length	Age-related hearing impairment	144	IVW	0.9969 (0.9763–1.0178)	0.767	0.913
Leukocyte telomere length	Age-related hearing impairment	144	MR Egger	0.9671 (0.9328–1.0026)	0.071	0.721
Leukocyte telomere length	Age-related hearing impairment	144	Weighted median	0.9857 (0.9584–1.0137)	0.313	0.694
Leukocyte telomere length	Age-related hearing impairment	144	Weighted mode	0.9721 (0.9429–1.0021)	0.071	0.826
Leukocyte telomere length	Conductive hearing loss, unspecified	158	IVW	1.1289 (0.9378–1.3589)	0.2	0.801
Leukocyte telomere length	Conductive hearing loss, unspecified	158	MR Egger	1.4071 (1.0263–1.9293)	0.035	0.721
Leukocyte telomere length	Conductive hearing loss, unspecified	158	Weighted median	1.3446 (0.9919–1.8228)	0.056	0.629
Leukocyte telomere length	Conductive hearing loss, unspecified	158	Weighted mode	1.4518 (0.9041–2.3313)	0.125	0.826
Leukocyte telomere length	Mixed conductive and sensorineural hearing loss	158	IVW	1.2082 (1.028–1.42)	0.022	0.506
Leukocyte telomere length	Mixed conductive and sensorineural hearing loss	158	MR Egger	1.1914 (0.903–1.5719)	0.217	0.779
Leukocyte telomere length	Mixed conductive and sensorineural hearing loss	158	Weighted median	1.1833 (0.9163–1.5281)	0.197	0.629
Leukocyte telomere length	Mixed conductive and sensorineural hearing loss	158	Weighted mode	1.1943 (0.8623–1.6542)	0.287	0.826
Leukocyte telomere length	Sensorineural hearing loss	158	IVW	1.0229 (0.9549–1.0957)	0.519	0.908
Leukocyte telomere length	Sensorineural hearing loss	158	MR Egger	1.1216 (0.9981–1.2605)	0.056	0.721
Leukocyte telomere length	Sensorineural hearing loss	158	Weighted median	1.0931 (1.0033–1.191)	0.042	0.629
Leukocyte telomere length	Sensorineural hearing loss	158	Weighted mode	1.1105 (1.0103–1.2207)	0.031	0.826
Leukocyte telomere length	Sudden idiopathic hearing loss	158	IVW	1.071 (0.8942–1.2829)	0.456	0.89
Leukocyte telomere length	Sudden idiopathic hearing loss	158	MR Egger	1.2754 (0.9374–1.7352)	0.124	0.721
Leukocyte telomere length	Sudden idiopathic hearing loss	158	Weighted median	1.1723 (0.8859–1.5511)	0.266	0.694
Leukocyte telomere length	Sudden idiopathic hearing loss	158	Weighted mode	1.3018 (0.8702–1.9476)	0.201	0.826
Lymphocyte telomere length	Age-related hearing impairment	5	IVW	0.9833 (0.9626–1.0044)	0.121	0.801
Lymphocyte telomere length	Age-related hearing impairment	5	MR Egger	1.7467 (0.0452–67.5363)	0.784	0.915
Lymphocyte telomere length	Age-related hearing impairment	5	Weighted median	0.9872 (0.9673–1.0076)	0.217	0.633
Lymphocyte telomere length	Age-related hearing impairment	5	Weighted mode	0.9862 (0.9642–1.0087)	0.294	0.826
Lymphocyte telomere length	Conductive hearing loss, unspecified	5	IVW	0.9046 (0.7652–1.0694)	0.24	0.833
Lymphocyte telomere length	Conductive hearing loss, unspecified	5	MR Egger	2.7e-05 (2.6e-16‒2844958.5481)	0.476	0.84
Lymphocyte telomere length	Conductive hearing loss, unspecified	5	Weighted median	0.8548 (0.6929–1.0546)	0.143	0.629
Lymphocyte telomere length	Conductive hearing loss, unspecified	5	Weighted mode	0.8168 (0.6168–1.0816)	0.231	0.826
Lymphocyte telomere length	Mixed conductive and sensorineural hearing loss	5	IVW	0.8754 (0.7623–1.0053)	0.059	0.716
Lymphocyte telomere length	Mixed conductive and sensorineural hearing loss	5	MR Egger	0.0011 (8.6e-13‒1467142.5352)	0.571	0.84
Lymphocyte telomere length	Mixed conductive and sensorineural hearing loss	5	Weighted median	0.8531 (0.7143–1.019)	0.08	0.629
Lymphocyte telomere length	Mixed conductive and sensorineural hearing loss	5	Weighted mode	0.8598 (0.6949–1.0637)	0.236	0.826
Lymphocyte telomere length	Sensorineural hearing loss	5	IVW	0.9673 (0.9208–1.0161)	0.185	0.801
Lymphocyte telomere length	Sensorineural hearing loss	5	MR Egger	2.5155 (0.0014–4446.2747)	0.825	0.931
Lymphocyte telomere length	Sensorineural hearing loss	5	Weighted median	0.9702 (0.9145–1.0294)	0.317	0.694
Lymphocyte telomere length	Sensorineural hearing loss	5	Weighted mode	0.9828 (0.9088–1.0628)	0.686	0.889
Lymphocyte telomere length	Sudden idiopathic hearing loss	5	IVW	1.0518 (0.9017–1.2269)	0.52	0.908
Lymphocyte telomere length	Sudden idiopathic hearing loss	5	MR Egger	2.8663 (6.7e-11‒121787512388.166)	0.938	0.954
Lymphocyte telomere length	Sudden idiopathic hearing loss	5	Weighted median	1.0438 (0.8616–1.2644)	0.662	0.949
Lymphocyte telomere length	Sudden idiopathic hearing loss	5	Weighted mode	1.0652 (0.8035–1.4122)	0.683	0.889
Memory T-cell telomere length	Age-related hearing impairment	5	IVW	1.0037 (0.9655–1.0434)	0.852	0.947
Memory T-cell telomere length	Age-related hearing impairment	5	MR Egger	0.9222 (0.4235–2.0084)	0.851	0.945
Memory T-cell telomere length	Age-related hearing impairment	5	Weighted median	0.9974 (0.9782–1.017)	0.794	0.949
Memory T-cell telomere length	Age-related hearing impairment	5	Weighted mode	0.9959 (0.9782–1.0139)	0.677	0.889
Memory T-cell telomere length	Conductive hearing loss, unspecified	5	IVW	0.961 (0.7769–1.1887)	0.714	0.913
Memory T-cell telomere length	Conductive hearing loss, unspecified	5	MR Egger	5.2864 (0.124–225.3404)	0.448	0.84
Memory T-cell telomere length	Conductive hearing loss, unspecified	5	Weighted median	0.9798 (0.7848–1.2232)	0.857	0.966
Memory T-cell telomere length	Conductive hearing loss, unspecified	5	Weighted mode	1.0088 (0.7967–1.2774)	0.946	0.97
Memory T-cell telomere length	Mixed conductive and sensorineural hearing loss	5	IVW	1.0004 (0.7752–1.2909)	0.998	0.998
Memory T-cell telomere length	Mixed conductive and sensorineural hearing loss	5	MR Egger	0.1833 (0.0017–19.7374)	0.529	0.84
Memory T-cell telomere length	Mixed conductive and sensorineural hearing loss	5	Weighted median	0.9104 (0.7499–1.1054)	0.343	0.719
Memory T-cell telomere length	Mixed conductive and sensorineural hearing loss	5	Weighted mode	0.8933 (0.7319–1.0904)	0.33	0.826
Memory T-cell telomere length	Sensorineural hearing loss	5	IVW	0.9544 (0.8766–1.0391)	0.282	0.833
Memory T-cell telomere length	Sensorineural hearing loss	5	MR Egger	0.7061 (0.1352–3.6872)	0.708	0.915
Memory T-cell telomere length	Sensorineural hearing loss	5	Weighted median	0.9423 (0.8826–1.006)	0.075	0.629
Memory T-cell telomere length	Sensorineural hearing loss	5	Weighted mode	0.9419 (0.8767–1.0118)	0.177	0.826
Memory T-cell telomere length	Sudden idiopathic hearing loss	5	IVW	0.8989 (0.7634–1.0584)	0.201	0.801
Memory T-cell telomere length	Sudden idiopathic hearing loss	5	MR Egger	0.4901 (0.0295–8.1314)	0.653	0.896
Memory T-cell telomere length	Sudden idiopathic hearing loss	5	Weighted median	0.8786 (0.721–1.0708)	0.2	0.629
Memory T-cell telomere length	Sudden idiopathic hearing loss	5	Weighted mode	0.879 (0.7129–1.0838)	0.294	0.826
Naive T-cell telomere length	Age-related hearing impairment	9	IVW	0.9947 (0.9838–1.0057)	0.345	0.833
Naive T-cell telomere length	Age-related hearing impairment	9	MR Egger	1.0498 (0.9333–1.1809)	0.445	0.84
Naive T-cell telomere length	Age-related hearing impairment	9	Weighted median	0.9975 (0.9828–1.0125)	0.743	0.949
Naive T-cell telomere length	Age-related hearing impairment	9	Weighted mode	1.0039 (0.9818–1.0266)	0.741	0.891
Naive T-cell telomere length	Conductive hearing loss, unspecified	9	IVW	0.9737 (0.8385–1.1306)	0.726	0.913
Naive T-cell telomere length	Conductive hearing loss, unspecified	9	MR Egger	0.4077 (0.0836–1.9888)	0.304	0.818
Naive T-cell telomere length	Conductive hearing loss, unspecified	9	Weighted median	0.9081 (0.7607–1.084)	0.286	0.694
Naive T-cell telomere length	Conductive hearing loss, unspecified	9	Weighted mode	0.8614 (0.6728–1.1028)	0.271	0.826
Naive T-cell telomere length	Mixed conductive and sensorineural hearing loss	9	IVW	0.9187 (0.8313–1.0152)	0.096	0.801
Naive T-cell telomere length	Mixed conductive and sensorineural hearing loss	9	MR Egger	0.8498 (0.2915–2.4768)	0.774	0.915
Naive T-cell telomere length	Mixed conductive and sensorineural hearing loss	9	Weighted median	0.8974 (0.7889–1.0209)	0.1	0.629
Naive T-cell telomere length	Mixed conductive and sensorineural hearing loss	9	Weighted mode	0.9029 (0.7737–1.0538)	0.231	0.826
Naive T-cell telomere length	Sensorineural hearing loss	9	IVW	1.0173 (0.9817–1.0541)	0.345	0.833
Naive T-cell telomere length	Sensorineural hearing loss	9	MR Egger	0.8579 (0.5859–1.256)	0.456	0.84
Naive T-cell telomere length	Sensorineural hearing loss	9	Weighted median	1.0262 (0.9832–1.0711)	0.236	0.661
Naive T-cell telomere length	Sensorineural hearing loss	9	Weighted mode	1.0207 (0.9642–1.0805)	0.501	0.889
Naive T-cell telomere length	Sudden idiopathic hearing loss	9	IVW	1.0441 (0.9343–1.167)	0.446	0.89
Naive T-cell telomere length	Sudden idiopathic hearing loss	9	MR Egger	1.5786 (0.4792–5.2001)	0.477	0.84
Naive T-cell telomere length	Sudden idiopathic hearing loss	9	Weighted median	1.0314 (0.8892–1.1962)	0.683	0.949
Naive T-cell telomere length	Sudden idiopathic hearing loss	9	Weighted mode	0.979 (0.7979–1.2011)	0.844	0.936
NK-cell telomere length	Age-related hearing impairment	4	IVW	1.0001 (0.9774–1.0233)	0.994	0.998
NK-cell telomere length	Age-related hearing impairment	4	MR Egger	1.3442 (0.9738–1.8555)	0.214	0.779
NK-cell telomere length	Age-related hearing impairment	4	Weighted median	1.011 (0.9881–1.0344)	0.349	0.719
NK-cell telomere length	Age-related hearing impairment	4	Weighted mode	1.0146 (0.9831–1.0472)	0.434	0.889
NK-cell telomere length	Conductive hearing loss, unspecified	4	IVW	1.0037 (0.836–1.205)	0.968	0.998
NK-cell telomere length	Conductive hearing loss, unspecified	4	MR Egger	1.1234 (0.0341–37.0285)	0.954	0.954
NK-cell telomere length	Conductive hearing loss, unspecified	4	Weighted median	1.0597 (0.8383–1.3396)	0.628	0.949
NK-cell telomere length	Conductive hearing loss, unspecified	4	Weighted mode	1.105 (0.8229–1.4836)	0.554	0.889
NK-cell telomere length	Mixed conductive and sensorineural hearing loss	4	IVW	1.0003 (0.8139–1.2295)	0.997	0.998
NK-cell telomere length	Mixed conductive and sensorineural hearing loss	4	MR Egger	0.2152 (0.0032–14.3571)	0.548	0.84
NK-cell telomere length	Mixed conductive and sensorineural hearing loss	4	Weighted median	1.0129 (0.831–1.2347)	0.899	0.968
NK-cell telomere length	Mixed conductive and sensorineural hearing loss	4	Weighted mode	1.0421 (0.8154–1.3317)	0.763	0.891
NK-cell telomere length	Sensorineural hearing loss	4	IVW	1.0379 (0.9335–1.154)	0.491	0.908
NK-cell telomere length	Sensorineural hearing loss	4	MR Egger	0.4681 (0.0543–4.0388)	0.561	0.84
NK-cell telomere length	Sensorineural hearing loss	4	Weighted median	1.0139 (0.9383–1.0955)	0.727	0.949
NK-cell telomere length	Sensorineural hearing loss	4	Weighted mode	0.9944 (0.8783–1.1258)	0.935	0.97
NK-cell telomere length	Sudden idiopathic hearing loss	4	IVW	0.9625 (0.8136–1.1387)	0.656	0.913
NK-cell telomere length	Sudden idiopathic hearing loss	4	MR Egger	1.2602 (0.0549–28.926)	0.898	0.946
NK-cell telomere length	Sudden idiopathic hearing loss	4	Weighted median	0.9934 (0.8096–1.2189)	0.949	0.977
NK-cell telomere length	Sudden idiopathic hearing loss	4	Weighted mode	1.0054 (0.7772–1.3006)	0.97	0.97
Pai1	Age-related hearing impairment	5	IVW	1.000001 (0.999992–1.00001)	0.877	0.959
Pai1	Age-related hearing impairment	5	MR Egger	1.000011 (0.999991–1.000031)	0.376	0.84
Pai1	Age-related hearing impairment	5	Weighted median	1.000004 (0.999993–1.000014)	0.518	0.886
Pai1	Age-related hearing impairment	5	Weighted mode	1.000004 (0.999991–1.000017)	0.556	0.889
Pai1	Conductive hearing loss, unspecified	5	IVW	1.0001 (0.9999–1.0003)	0.578	0.913
Pai1	Conductive hearing loss, unspecified	5	MR Egger	0.9997 (0.9994–1.0001)	0.222	0.779
Pai1	Conductive hearing loss, unspecified	5	Weighted median	0.9999 (0.9997–1.0001)	0.47	0.843
Pai1	Conductive hearing loss, unspecified	5	Weighted mode	0.9999 (0.9996–1.0002)	0.512	0.889
Pai1	Mixed conductive and sensorineural hearing loss	5	IVW	0.999961 (0.9998–1.0001)	0.503	0.908
Pai1	Mixed conductive and sensorineural hearing loss	5	MR Egger	0.9997 (0.9995–1.000003)	0.148	0.747
Pai1	Mixed conductive and sensorineural hearing loss	5	Weighted median	1.000013 (0.9999–1.0002)	0.869	0.966
Pai1	Mixed conductive and sensorineural hearing loss	5	Weighted mode	0.9999 (0.9997–1.0001)	0.226	0.826
Pai1	Sensorineural hearing loss	5	IVW	0.999978 (0.9999–1.000023)	0.328	0.833
Pai1	Sensorineural hearing loss	5	MR Egger	0.9999 (0.9998‒0.99999)	0.118	0.721
Pai1	Sensorineural hearing loss	5	Weighted median	0.999959 (0.9999–1.000006)	0.085	0.629
Pai1	Sensorineural hearing loss	5	Weighted mode	0.999963 (0.9999–1.000015)	0.234	0.826
Pai1	Sudden idiopathic hearing loss	5	IVW	1.000028 (0.9999–1.0002)	0.663	0.913
Pai1	Sudden idiopathic hearing loss	5	MR Egger	0.9998 (0.9995–1.0001)	0.285	0.818
Pai1	Sudden idiopathic hearing loss	5	Weighted median	0.999988 (0.9998–1.0001)	0.883	0.966
Pai1	Sudden idiopathic hearing loss	5	Weighted mode	0.999983 (0.9998–1.0002)	0.852	0.936
Phenoage	Age-related hearing impairment	11	IVW	1.0014 (0.9969–1.0059)	0.536	0.908
Phenoage	Age-related hearing impairment	11	MR Egger	1.0076 (0.997–1.0182)	0.194	0.779
Phenoage	Age-related hearing impairment	11	Weighted median	0.9986 (0.9923–1.005)	0.671	0.949
Phenoage	Age-related hearing impairment	11	Weighted mode	0.9973 (0.9869–1.0077)	0.617	0.889
Phenoage	Conductive hearing loss, unspecified	10	IVW	0.9696 (0.9153–1.027)	0.292	0.833
Phenoage	Conductive hearing loss, unspecified	10	MR Egger	0.8885 (0.724–1.0903)	0.29	0.818
Phenoage	Conductive hearing loss, unspecified	10	Weighted median	0.9832 (0.9131–1.0588)	0.654	0.949
Phenoage	Conductive hearing loss, unspecified	10	Weighted mode	1.0264 (0.9122–1.1549)	0.675	0.889
Phenoage	Mixed conductive and sensorineural hearing loss	10	IVW	0.994 (0.9483–1.0419)	0.803	0.937
Phenoage	Mixed conductive and sensorineural hearing loss	10	MR Egger	0.9739 (0.8253–1.1493)	0.763	0.915
Phenoage	Mixed conductive and sensorineural hearing loss	10	Weighted median	1.0009 (0.9426–1.0628)	0.977	0.977
Phenoage	Mixed conductive and sensorineural hearing loss	10	Weighted mode	0.9967 (0.9191–1.0809)	0.939	0.97
Phenoage	Sensorineural hearing loss	10	IVW	0.9895 (0.9723–1.0069)	0.236	0.833
Phenoage	Sensorineural hearing loss	10	MR Egger	1.0041 (0.9414–1.0709)	0.905	0.946
Phenoage	Sensorineural hearing loss	10	Weighted median	0.9953 (0.973–1.0182)	0.686	0.949
Phenoage	Sensorineural hearing loss	10	Weighted mode	1.0096 (0.9733–1.0472)	0.622	0.889
Phenoage	Sudden idiopathic hearing loss	10	IVW	1.0304 (0.9779–1.0858)	0.262	0.833
Phenoage	Sudden idiopathic hearing loss	10	MR Egger	1.0727 (0.8923–1.2896)	0.476	0.84
Phenoage	Sudden idiopathic hearing loss	10	Weighted median	1.0267 (0.9581–1.1003)	0.455	0.837
Phenoage	Sudden idiopathic hearing loss	10	Weighted mode	1.0288 (0.9295–1.1387)	0.597	0.889
Telomere length	Age-related hearing impairment	130	IVW	0.9945 (0.9735–1.016)	0.613	0.913
Telomere length	Age-related hearing impairment	130	MR Egger	0.9635 (0.9285‒0.9998)	0.051	0.721
Telomere length	Age-related hearing impairment	130	Weighted median	0.9815 (0.9544–1.0094)	0.192	0.629
Telomere length	Age-related hearing impairment	130	Weighted mode	0.9754 (0.9468–1.0048)	0.102	0.826
Telomere length	Conductive hearing loss, unspecified	133	IVW	1.1427 (0.9372–1.3932)	0.187	0.801
Telomere length	Conductive hearing loss, unspecified	133	MR Egger	1.4803 (1.0442–2.0986)	0.029	0.721
Telomere length	Conductive hearing loss, unspecified	133	Weighted median	1.3327 (0.9407–1.8878)	0.106	0.629
Telomere length	Conductive hearing loss, unspecified	133	Weighted mode	1.1759 (0.766–1.8049)	0.46	0.889
Telomere length	Mixed conductive and sensorineural hearing loss	133	IVW	1.1324 (0.9505–1.3491)	0.164	0.801
Telomere length	Mixed conductive and sensorineural hearing loss	133	MR Egger	1.1608 (0.8498–1.5856)	0.35	0.84
Telomere length	Mixed conductive and sensorineural hearing loss	133	Weighted median	1.2898 (0.9952–1.6717)	0.054	0.629
Telomere length	Mixed conductive and sensorineural hearing loss	133	Weighted mode	1.1429 (0.8684–1.5041)	0.342	0.826
Telomere length	Sensorineural hearing loss	133	IVW	1.0021 (0.9285–1.0815)	0.957	0.998
Telomere length	Sensorineural hearing loss	133	MR Egger	1.1125 (0.9727–1.2723)	0.122	0.721
Telomere length	Sensorineural hearing loss	133	Weighted median	1.0699 (0.9746–1.1744)	0.156	0.629
Telomere length	Sensorineural hearing loss	133	Weighted mode	1.0947 (0.9893–1.2113)	0.082	0.826
Telomere length	Sudden idiopathic hearing loss	133	IVW	1.0741 (0.8921–1.2933)	0.45	0.89
Telomere length	Sudden idiopathic hearing loss	133	MR Egger	1.2047 (0.866–1.6757)	0.271	0.818
Telomere length	Sudden idiopathic hearing loss	133	Weighted median	1.2144 (0.8983–1.6417)	0.207	0.629
Telomere length	Sudden idiopathic hearing loss	133	Weighted mode	1.303 (0.9217–1.8422)	0.136	0.826

Notes: This table presents the main results of the two-sample Mendelian randomization analysis using the inverse-variance weighted (IVW), MR-Egger, weighted median, and weighted mode methods. These results are from the primary analysis before any outlier correction was applied. Results are presented as Odds Ratios (OR) with 95% Confidence Intervals (95% CI).

MR, Mendelian Randomization; IVW, Inverse-Variance Weighted; OR, Odds Ratio; CI, Confidence Interval; p, p-value; N.SNPs, Number of SNPs used as instrumental variables.

### Sensitivity analyses

We performed a comprehensive suite of sensitivity analyses to evaluate the consistency and stability of our findings (Fig. 3). While evidence for heterogeneity was detected by Cochran’s *Q*-test in several analyses (Supplementary Table 3), our primary random-effects IVW model was chosen to account for this. The MR Egger intercept test was not significant for most analyses, except for the association of leukocyte telomere length and age-related hearing impairment (p = 0.046), and total telomere length with age-related hearing impairment (p = 0.043), suggesting possible directional pleiotropy in these specific analyses (Supplementary Table 3).

**Fig. 3:**
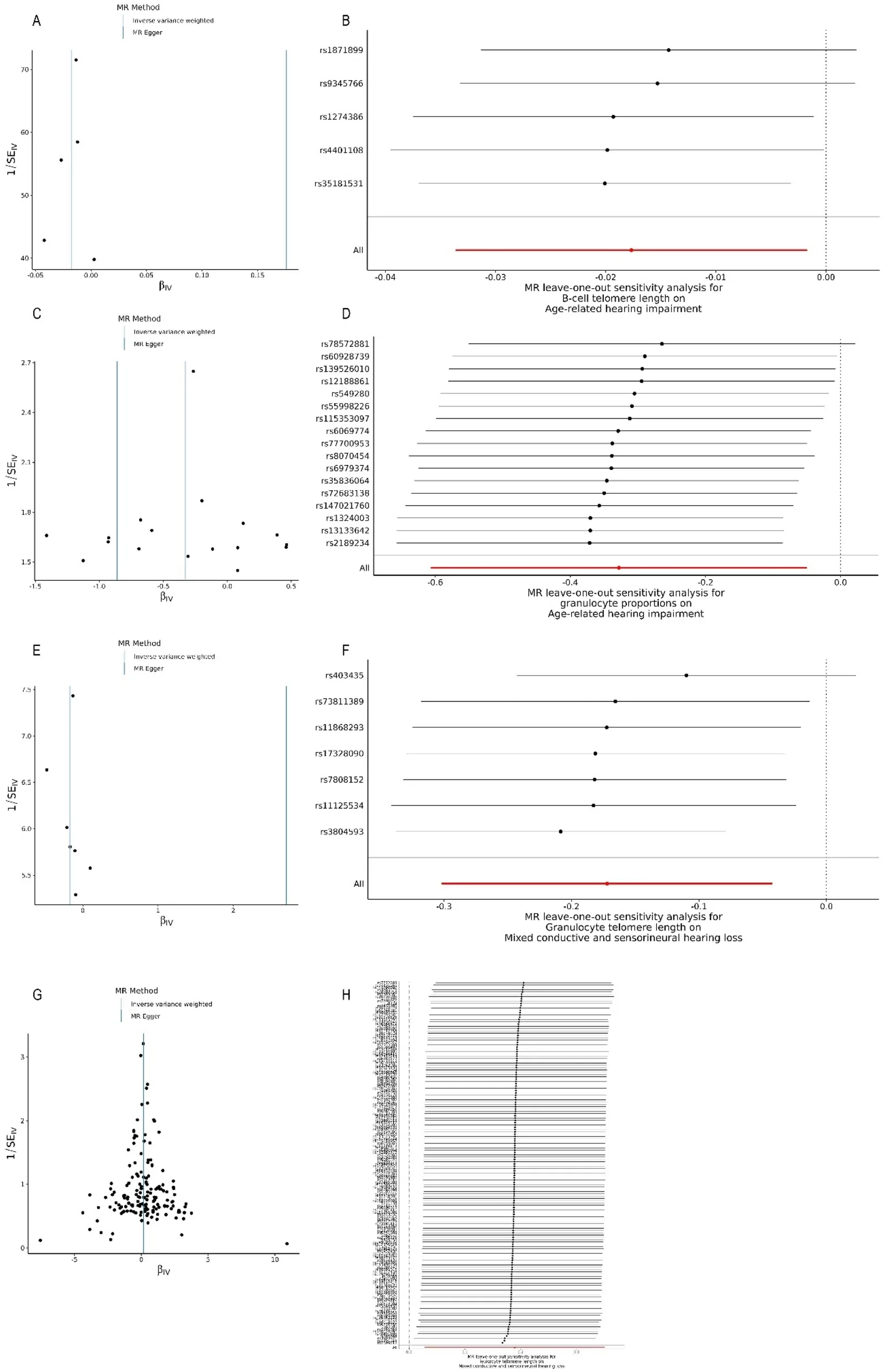
Primary analysis ‒ sensitivity plots. (A) Funnel plot to assess heterogeneity and pleiotropy for the association of B-cell telomere length with age-related hearing impairment. (B) Leave-one-out sensitivity analysis for the association of B-cell telomere length with age-related hearing impairment. (C) Funnel plot to assess heterogeneity and pleiotropy for the association of granulocyte proportions with age-related hearing impairment. (D) Leave-one-out sensitivity analysis for the association of granulocyte proportions with age-related hearing impairment. (E) Funnel plot to assess heterogeneity and pleiotropy for the association of granulocyte telomere length with mixed hearing loss. (F) Leave-one-out sensitivity analysis for the association of granulocyte telomere length with mixed hearing loss. (G) Funnel plot to assess heterogeneity and pleiotropy for the association of leukocyte telomere length with mixed hearing loss. (H) Leave-one-out sensitivity analysis for the association of leukocyte telomere length with mixed hearing loss.

The MR-PRESSO analysis was conducted to identify and correct for potential outliers (Supplementary Table 4). The primary four nominal associations remained generally consistent after this correction. The analysis also identified two potential associations that emerged after the removal of specific outlier SNPs. A suggestive association was observed between GrimAge and sudden idiopathic hearing loss (OR = 1.06, 95% CI 1.00–1.13, p = 0.038, FDR_p = 0.521) and between leukocyte telomere length and sensorineural hearing loss (OR = 1.06, 95% CI 1.00–1.12, p = 0.045, FDR_p = 0.521) (Figs. 4,5, Supplementary Table 5). As these associations were only apparent post-correction, they should be interpreted with caution. Overall, the outlier correction process supported the stability of the reported nominal associations. The final MR-PRESSO analysis did not identify additional outliers (Supplementary Table 6), and updated sensitivity analyses did not indicate substantial horizontal pleiotropy across all reported associations (Table 2), supporting the stability of our final models.

**Fig. 4:**
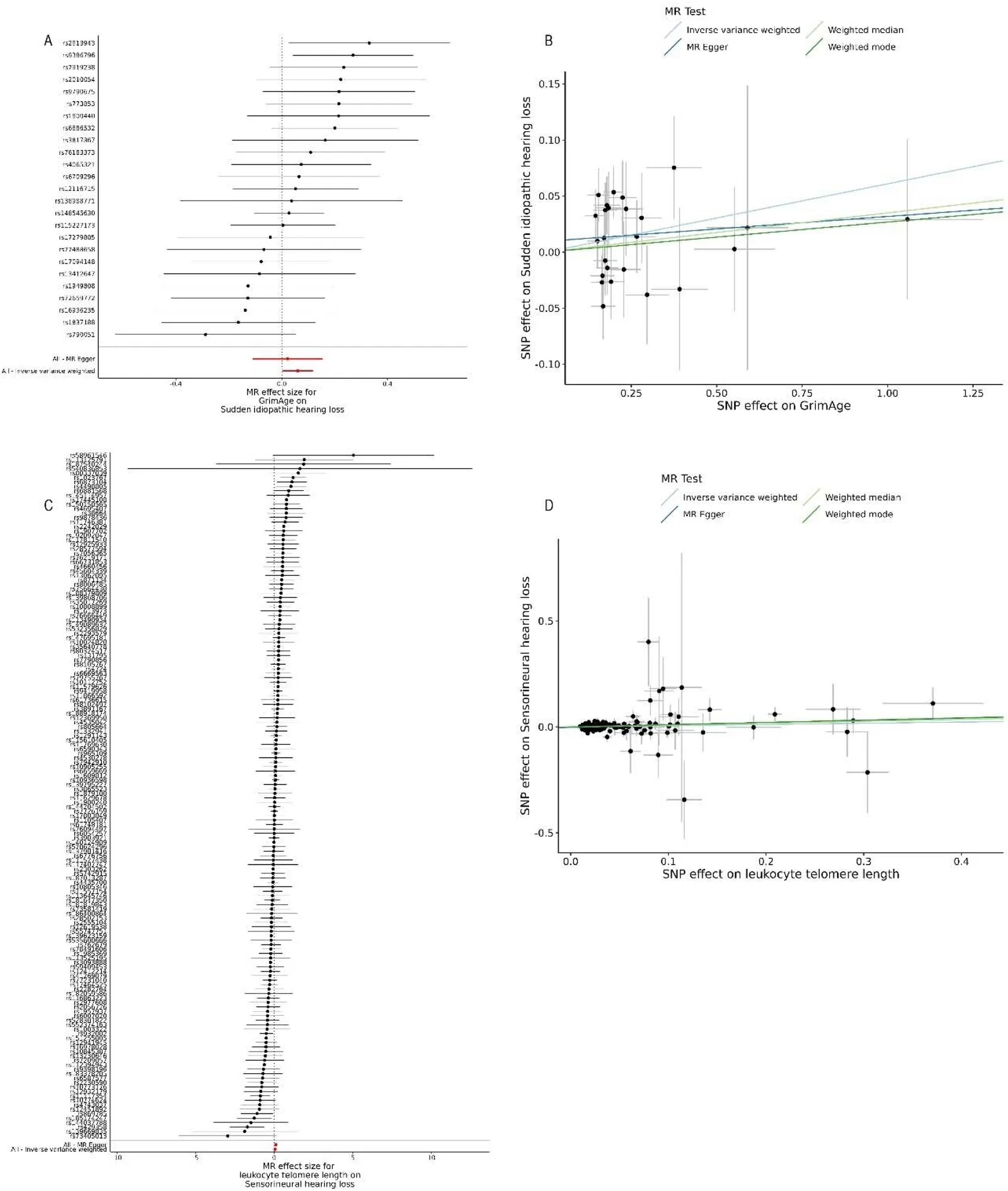
Post-outlier removal ‒ causal effect plots. (A) Forest plot for GrimAge and sudden idiopathic hearing loss after outlier removal. (B) Scatter plot for GrimAge and sudden idiopathic hearing loss after outlier removal. (C) Forest plot for leukocyte telomere length and sensorineural hearing loss after outlier removal. (D) Scatter plot for leukocyte telomere length and sensorineural hearing loss after outlier removal.

**Fig. 5:**
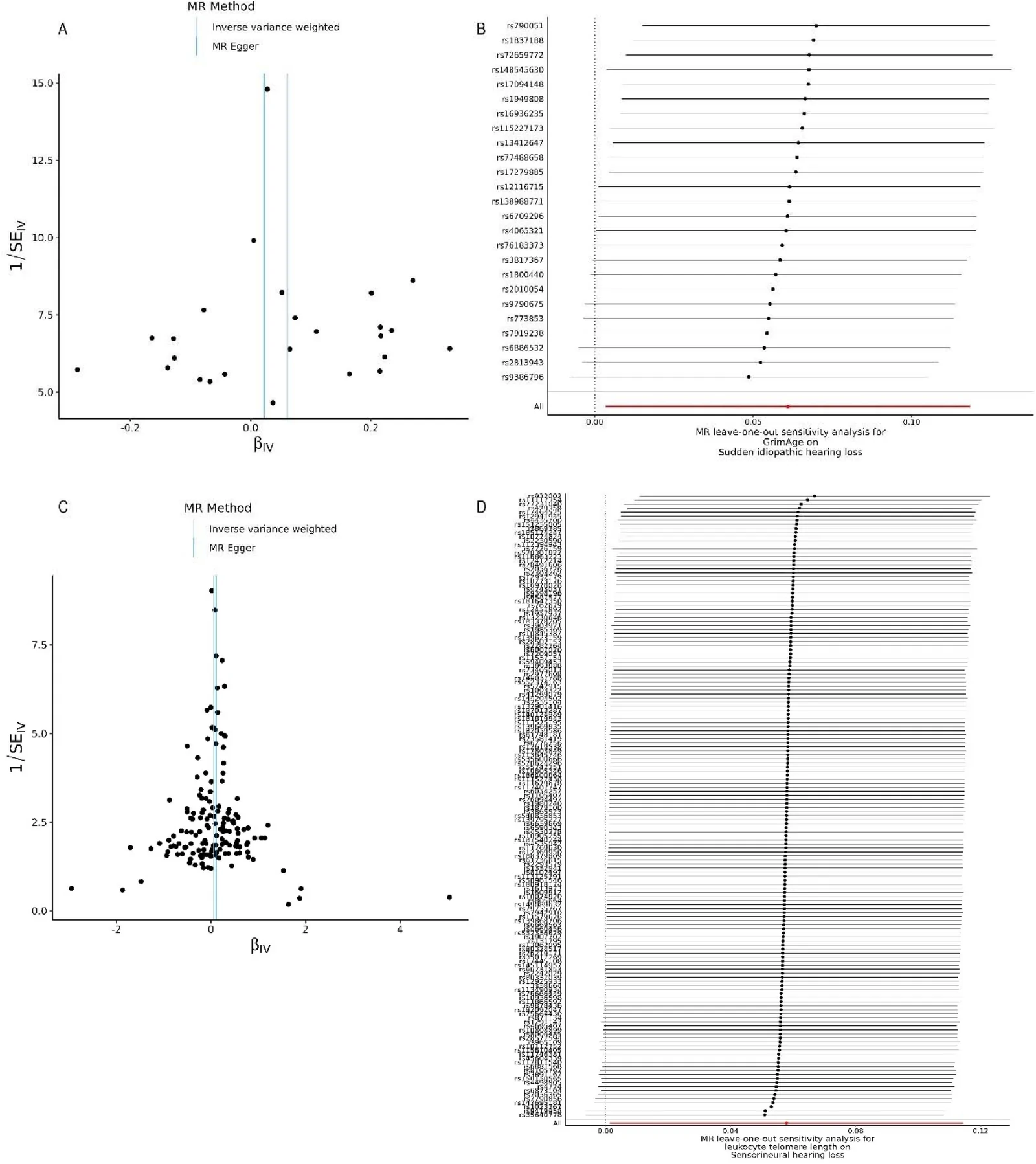
Post-outlier removal - sensitivity plots. (A) Funnel plot for GrimAge and sudden idiopathic hearing loss after outlier removal. (B) Leave-one-out analysis for GrimAge and sudden idiopathic hearing loss after outlier removal. (C) Funnel plot for leukocyte telomere length and sensorineural hearing loss after outlier removal. (D) Leave-one-out analysis for leukocyte telomere length and sensorineural hearing loss after outlier removal.

**Table 2:** Results of heterogeneity and pleiotropy tests after MR-PRESSO outlier removal.

Exposure	Outcome	Heterogeneity		Pleiotropy	
		Q statistic (IVW)	p-value	MR-Egger Intercept	p-value
B-cell telomere length	Sudden idiopathic hearing loss	7566	0.109	0.777	0.184
B-cell telomere length	Conductive hearing loss, unspecified	6004	0.199	0.289	0.659
B-cell telomere length	Age-related hearing impairment	2213	0.697	−0.031	0.504
B-cell telomere length	Sensorineural hearing loss	4076	0.396	0.098	0.527
B-cell telomere length	Mixed conductive and sensorineural hearing loss	2835	0.586	−0.419	0.328
granulocyte proportions	Sudden idiopathic hearing loss	22,365	0.132	−0.007	0.801
granulocyte proportions	Conductive hearing loss, unspecified	17,169	0.375	−0.015	0.553
granulocyte proportions	Age-related hearing impairment	13,702	0.621	0.003	0.18
granulocyte proportions	Sensorineural hearing loss	23,586	0.099	−0.004	0.662
granulocyte proportions	Mixed conductive and sensorineural hearing loss	23,228	0.079	0.005	0.854
Granulocyte telomere length	Sudden idiopathic hearing loss	4904	0.556	0.062	0.841
Granulocyte telomere length	Conductive hearing loss, unspecified	4,34	0.631	−0.063	0.852
Granulocyte telomere length	Age-related hearing impairment	5151	0.525	0.016	0.6
Granulocyte telomere length	Sensorineural hearing loss	8035	0.236	0.158	0.163
Granulocyte telomere length	Mixed conductive and sensorineural hearing loss	6877	0.332	−0.462	0.142
GrimAge	Sudden idiopathic hearing loss	27,277	0.292	0.01	0.532
GrimAge	Conductive hearing loss, unspecified	4079	0.024	0.015	0.453
GrimAge	Age-related hearing impairment	26,986	0.357	−7,40E-04	0.672
GrimAge	Sensorineural hearing loss	45,938	0.007	0.01	0.095
GrimAge	Mixed conductive and sensorineural hearing loss	20,731	0.708	0.019	0.134
HannumAge	Sudden idiopathic hearing loss	9474	0.304	−0.029	0.51
HannumAge	Conductive hearing loss, unspecified	10,161	0.254	0.028	0.578
HannumAge	Age-related hearing impairment	3615	0.729	−0.005	0.276
HannumAge	Sensorineural hearing loss	6737	0.565	0.004	0.744
HannumAge	Mixed conductive and sensorineural hearing loss	14,704	0.065	0.054	0.257
HorvathAge	Sudden idiopathic hearing loss	21,407	0.556	0.011	0.421
HorvathAge	Conductive hearing loss, unspecified	25,516	0.324	−0.018	0.244
HorvathAge	Age-related hearing impairment	29,259	0.138	−4,20E-04	0.805
HorvathAge	Sensorineural hearing loss	30,809	0.128	−0.007	0.164
HorvathAge	Mixed conductive and sensorineural hearing loss	9654	0.993	−0.007	0.552
leukocyte telomere length	Sudden idiopathic hearing loss	189,316	0.04	−0.006	0.173
leukocyte telomere length	Conductive hearing loss, unspecified	168,281	0.255	−0.008	0.094
leukocyte telomere length	Age-related hearing impairment	166,453	0.063	9,30E-04	0.056
leukocyte telomere length	Sensorineural hearing loss	173,027	0.128	−0.002	0.198
leukocyte telomere length	Mixed conductive and sensorineural hearing loss	176,055	0.13	0.002	0.666
Lymphocyte telomere length	Sudden idiopathic hearing loss	3297	0.509	−0.154	0.941
Lymphocyte telomere length	Conductive hearing loss, unspecified	3071	0.546	1605	0.48
Lymphocyte telomere length	Age-related hearing impairment	8,54	0.074	−0.089	0.778
Lymphocyte telomere length	Sensorineural hearing loss	0.579	0.965	−0.147	0.818
Lymphocyte telomere length	Mixed conductive and sensorineural hearing loss	3118	0.538	1026	0.578
Memory T-cell telomere length	Sudden idiopathic hearing loss	1462	0.833	0.098	0.7
Memory T-cell telomere length	Conductive hearing loss, unspecified	5747	0.219	−0.275	0.438
Memory T-cell telomere length	Age-related hearing impairment	1281	0.734	−0.013	0.611
Memory T-cell telomere length	Sensorineural hearing loss	10,608	0.031	0.049	0.744
Memory T-cell telomere length	Mixed conductive and sensorineural hearing loss	0.611	0.894	−0.142	0.62
Naive T-cell telomere length	Sudden idiopathic hearing loss	4944	0.763	−0.066	0.517
Naive T-cell telomere length	Conductive hearing loss, unspecified	12,25	0.14	0.138	0.315
Naive T-cell telomere length	Age-related hearing impairment	8033	0.43	−0.009	0.397
Naive T-cell telomere length	Sensorineural hearing loss	3432	0.904	0.027	0.408
Naive T-cell telomere length	Mixed conductive and sensorineural hearing loss	4901	0.768	0.012	0.89
NK-cell telomere length	Sudden idiopathic hearing loss	1848	0.604	−0.043	0.881
NK-cell telomere length	Conductive hearing loss, unspecified	2109	0.55	−0.018	0.955
NK-cell telomere length	Age-related hearing impairment	5639	0.131	−0.047	0.214
NK-cell telomere length	Sensorineural hearing loss	2676	0.262	−0.093	0.673
NK-cell telomere length	Mixed conductive and sensorineural hearing loss	5596	0.133	0.247	0.547
PAI1	Sudden idiopathic hearing loss	3,88	0.422	0.167	0.198
PAI1	Conductive hearing loss, unspecified	9266	0.055	0.255	0.133
PAI1	Age-related hearing impairment	2283	0.684	−0.008	0.359
PAI1	Sensorineural hearing loss	5116	0.276	0.061	0.157
PAI1	Mixed conductive and sensorineural hearing loss	4197	0.38	0.166	0.166
PhenoAge	Sudden idiopathic hearing loss	5176	0.819	−0.015	0.667
PhenoAge	Conductive hearing loss, unspecified	9294	0.411	0.032	0.409
PhenoAge	Age-related hearing impairment	9834	0.455	−0.003	0.239
PhenoAge	Sensorineural hearing loss	9839	0.364	−0.005	0.655
PhenoAge	Mixed conductive and sensorineural hearing loss	3987	0.912	0.007	0.807
telomere length	Sudden idiopathic hearing loss	146,544	0.183	−0.004	0.411
telomere length	Conductive hearing loss, unspecified	141,498	0.27	−0.009	0.081
telomere length	Age-related hearing impairment	166,64	0.01	8,90E-04	0.077
telomere length	Sensorineural hearing loss	160,847	0.03	−0.002	0.219
telomere length	Mixed conductive and sensorineural hearing loss	150,578	0.116	4,00E-04	0.927

Notes: This table provides the results of the heterogeneity and pleiotropy tests conducted on the outlier-corrected datasets. Comparing these results with Table S5 allows for an assessment of whether removing outliers successfully reduced heterogeneity or altered the evidence for pleiotropy.

MR, Mendelian Randomization; IVW, Inverse-Variance Weighted.

## Discussion

In this comprehensive MR study, we examined potential associations between aging biomarkers and five subtypes of hearing impairment. Our founding’s suggested that these associations may vary by cell type, rather than simply reflecting a systemic aging process. A notable observation was the nominal and directionally opposing associations of telomere length with mixed hearing loss: longer granulocyte telomeres were associated with lower odds, whereas longer leukocyte telomeres were associated with higher odds. These results suggest the potential importance of immune cell specificity and support further investigation of specific pathways for future mechanistic research.

One of the most intriguing observations was the paradoxicaland cell-type-specific suggestive associations of telomere length with mixed hearing loss. Genetically predicted longer granulocyte telomere length was associated with higher odds. One possible explanation is that leukocyte telomere length represents a composite measure across heterogeneous immune-cell populations, whereas granulocyte telomere length reflects a more specific myeloid-cell compartment. Granulocytes are short-lived cells central to acute inflammatory responses, while the broader leukocyte pool includes longer-lived lymphocyte populations.^19^ Therefore, bulk leukocyte telomere length may partly obscure cell-specific biological signals. However, because these associations were nominal and did not survive FDR correction, alternative explanations, including phenotype heterogeneity, data noise, and residual horizontal pleiotropy, cannot be excluded. This finding should therefore be viewed as hypothesis-generating and requires validation in larger cell-resolved datasets.^20^

Equally paradoxical was the nominal association between a higher proportion of granulocytes and a reduced risk of age-related hearing impairment. This finding appears inconsistent with the conventional “inflammaging” hypothesis, which suggests that chronic, low-grade, neutrophil-related inflammation may contribute to age-related tissue damage, including cochlear injury.^21^ Several explanations are possible. First, genetically predicted granulocyte proportion may capture aspects of immune-cell composition or inflammatory responsiveness that are not equivalent to chronic inflammatory activation. Second, the association may reflect unmeasured horizontal pleiotropy, in which genetic variants related to granulocyte traits influence hearing through pathways independent of granulocyte biology. Third, diagnostic heterogeneity in broadly defined hearing impairment phenotypes may also contribute to unstable estimates. Therefore, this result should not be interpreted as evidence that granulocyte predominance is protective, but rather as a signal that immune-cell composition deserves further investigation in hearing aging.

Our analysis provides critical clarity on the causal role of epigenetic clocks, demonstrating that they are likely not universal drivers of hearing impairment but may instead signal risk for specific, acute subtypes like Sudden Idiopathic Hearing Loss (SIHL).^22^ This finding is highly significant, as it indicates that the broad associations reported in observational studies may be influenced by confounding rather than direct causality. From a clinical perspective, this suggests that general 'anti-aging' interventions targeting epigenetic clocks may be an inefficient strategy for preventing common hearing loss.

GrimAge is a unique clock that incorporates smoking pack-years and plasma proteins, making it a strong predictor of mortality and cardiovascular disease.^23^ The highly specific association we uncovered between GrimAge and SIHL represents a key hypothesis-generating result of our study. This finding is particularly compelling given that GrimAge uniquely integrates biomarkers of physiological stress and cardiovascular health, aligning perfectly with the suspected vascular etiology of SIHL.^24^ Rather than a fragile signal, we interpret this as a precise clue pointing towards a specific pathophysiology. This opens a vital new avenue for research, suggesting that the molecular components within GrimAge ‒ such as inflammatory proteins or smoking-related damage markers ‒ could be novel therapeutic targets or risk stratification tools for SIHL, a condition that currently lacks effective prevention strategies.

Our findings must be placed in the context of the existing literature. The lack of a robust causal link between overall telomere length and ARHI is consistent with previous MR studies, strengthening the conclusion that this widely used biomarker is likely not a major causal factor for common hearing loss.^25^^,^^26^ Our null results for most epigenetic clocks contrast with observational findings,^27^ but this is a common theme in MR research, which often refutes or qualifies associations that are susceptible to confounding in traditional study designs.^28^ The novelty of our work lies in the granular analysis of hearing subtypes and cell-specific markers, which, despite the largely null overarching conclusion, uncovers a layer of complexity previously unexamined.

These findings may also inform future precision-oriented research in hearing health. The observed cell-type-related patterns suggest that biological aging may influence hearing impairment through immune-cell-specific and inflammatory pathways rather than through a single systemic aging process. Future studies that combine aging biomarkers with audiometric profiles, longitudinal hearing trajectories, and immune or vascular indicators may help define biologically distinct subgroups of hearing impairment and support more individualized prevention strategies.

This study has several key strengths, including the two-sample MR design, the use of large-scale GWAS data, the systematic analysis of multiple hearing subtypes, and the novel inclusion of cell-specific telomere length data. However, several limitations must be acknowledged. First, our findings are restricted to individuals of European ancestry and may not be generalizable to other populations. Second, the statistical power to detect small effects, particularly for cell-specific telomere length (derived from n = 902) and less common hearing subtypes, was limited, raising the possibility of Type II error. Third, while we conducted extensive sensitivity analyses, the possibility of unmeasured horizontal pleiotropy cannot be fully excluded. Fourth, none of the nominal associations remained significant after FDR correction, indicating that these findings should be interpreted as suggestive and hypothesis-generating rather than definitive evidence. Finally, hearing impairment phenotypes in large consortia are often based on self-report or broad clinical codes, which may lack the precision of audiometric testing and introduce diagnostic noise.

In conclusion, our MR analysis provides suggestive genetic evidence for a complex, cell-type-specific relationship between aging biomarkers and hearing impairment. We observed nominal associations, including lower odds of mixed hearing loss with longer granulocyte telomere length and higher odds with longer leukocyte telomere length. These findings support further attention to distinct immune cell pathways beyond systemic aging markers. This study provides a hypothesis-generating basis for future research on cellular aging pathways relevant to hearing preservation.

## ORCID ID

Xiaojun Jiang: 0000-0002-4810-5608

Chen Tang: 0009-0002-2518-876X

Wei Yuan: 0009-0008-1236-8677

## Authors’ contributions

Xiaojun Jiang and Chen Tang carried out the studies, participated in collecting data, and drafted the manuscript. Wei Yuan performed the statistical analysis and participated in its design. Xiaojun Jiang and Chen Tang participated in acquisition, analysis, or interpretation of data and draft the manuscript. All authors read and approved the final manuscript.

## Funding

None.

## Data availability

All data generated or analyzed during this study are included in this article and supplementary information files.

## Declaration of competing interest

The authors declare no conflicts of interest.
